# Suboptimal hydration remodels metabolism, promotes degenerative diseases, and shortens life

**DOI:** 10.1172/jci.insight.130949

**Published:** 2019-09-05

**Authors:** Michele D. Allen, Danielle A. Springer, Maurice B. Burg, Manfred Boehm, Natalia I. Dmitrieva

**Affiliations:** 1 Murine Phenotyping Core,; 2 Renal Cellular and Molecular Biology Section, and; 3 Laboratory of Cardiovascular Regenerative Medicine, National Heart, Lung, and Blood Institute, Bethesda, Maryland, USA.

**Keywords:** Aging, Epidemiology

## Abstract

With increased life expectancy worldwide, there is an urgent need for improving preventive measures that delay the development of age-related degenerative diseases. Here, we report evidence from mouse and human studies that this goal can be achieved by maintaining optimal hydration throughout life. We demonstrate that restricting the amount of drinking water shortens mouse lifespan with no major warning signs up to 14 months of life, followed by sharp deterioration. Mechanistically, water restriction yields stable metabolism remodeling toward metabolic water production with greater food intake and energy expenditure, an elevation of markers of inflammation and coagulation, accelerated decline of neuromuscular coordination, renal glomerular injury, and the development of cardiac fibrosis. In humans, analysis of data from the Atherosclerosis Risk in Communities (ARIC) study revealed that hydration level, assessed at middle age by serum sodium concentration, is associated with markers of coagulation and inflammation and predicts the development of many age-related degenerative diseases 24 years later. The analysis estimates that improving hydration throughout life may greatly decrease the prevalence of degenerative diseases, with the most profound effect on dementia, heart failure (HF), and chronic lung disease (CLD), translating to the development of these diseases in 3 million fewer people in the United States alone.

## Abstract 2

In mice, restricting the amount of drinking water shortens lifespan and may accelerate degenerative changes leading to age-related chronic diseases.

## Graphical abstract

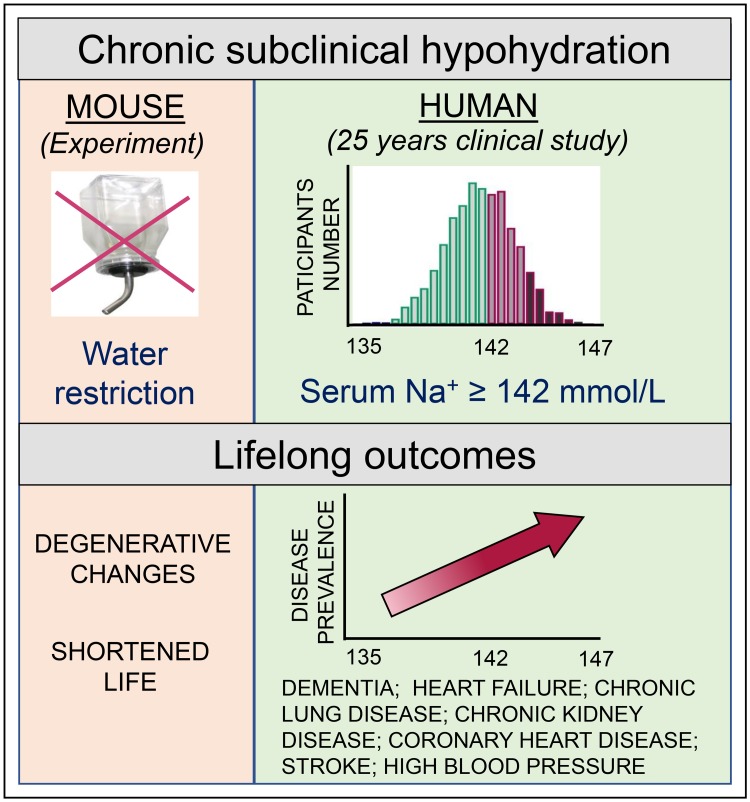

## Introduction

Because of great progress in the prevention and treatment of infectious diseases and technological advances that have improved life and work conditions, the world has seen a tremendous increase in life expectancy. With such achievements, aging of the world population creates challenges for medical researchers to find new ways to combat and care for age-dependent chronic disorders that account for a growing proportion of the global burden of disease (1). The solutions to these challenges include developing preventive measures that would prolong a healthy lifespan, finding new treatments, and improving age-appropriate care for the diseases that have already developed. Here, we provide evidence from mouse and human studies that maintaining optimal hydration throughout a person’s lifetime provides protection from the development of age-dependent chronic disorders.

Water is the main constituent of the body. It accounts for 75% and 55% of body weight in newborns and the elderly, respectively, and it is essential for homeostasis and life. Water balance, which is the balance between water intake and water output, is regulated relative to electrolyte content within 0.2% of body weight and keeps solutes concentrations (osmolality) within a normal reference range at 275–295 mosmol/kg and its principal determinant sodium at 135–145 mmol/L. To prevent dehydration, a complex network of hormonal and neuroregulatory mechanisms is activated in response to net water loss leading to intravascular volume depletion and increased osmolality above its osmotic “set-point” of 280–290 mosmol/kg. These mechanisms include stimulation of thirst to encourage water intake, increased water reabsorption by the kidneys to decrease water output, and stimulation of vasoconstriction to compensate for decreased intravascular volume mediated by arginine vasopressin (AVP) and renin-angiotensin-aldosterone system (RAAS) (2–9).

It is generally assumed that levels of hydration that keep serum osmolality and/or serum sodium within their reference ranges are considered safe. However, recent studies in cell culture and mouse models demonstrate that variations even within such a narrow range produce physiologically relevant and potentially pathological changes in vascular endothelial cells. Thus, increasing the concentration of extracellular sodium within 135–145 mmol/L toward the higher end of the reference range in cell culture leads to vascular endothelial cells activation characterized by the increased expression of mediators of coagulation and inflammation, such as vWF (10), VCAM-1, E-selectin, and MCP-1 (11, 12), reduced nitric oxide release (13), and a stiffened endothelial glycocalyx (14, 15). Similar changes in vascular endothelium occur in vivo in response to moderate water restriction that elevates mouse plasma sodium concentration by 5 mmol/L (10, 11). Therefore, based on the vascular effects of extracellular sodium and on well documented pathological consequences of RAAS overactivation (8, 16), we hypothesized that even subclinical mild hypohydration can potentially impact long-term health outcomes.

In our current study, we investigated the long-term impact of chronic subclinical hypohydration in mice and humans. The study provided evidence that subtle changes in metabolic and homeostatic programs elicited by changes in hydration levels within subclinical ranges had profound effects on long-term health outcomes. Using a mouse model of lifelong mild water restriction, we demonstrated that hypohydration led to an increased metabolic rate and energy expenditure and induced a low-grade proinflammatory and procoagulative state; these conditions are all known promoters of chronic morbidity, disability, and premature death (17–21). Consistently, water restriction caused accelerated renal, cardiac, and neuromotor degenerative changes and decreased mouse lifespan.

In humans, long-term hydration levels can be assessed by serum sodium concentration that has very little variability in individual persons over a long period of time (22, 23). By analyzing data from the Atherosclerosis Risk in Communities (ARIC) study, we showed that the outcomes of subclinical hypohydration in humans were strikingly similar. Serum sodium levels measured at middle age showed a positive association with markers of coagulation and inflammation and predicted the development of age-related chronic degenerative diseases 24 years later. By analyzing disease prevalence, we showed that a serum sodium level below 142 mmol/L greatly reduced the risk for the development of many degenerative diseases including heart failure (HF), dementia, and chronic lung disease (CLD). These findings indicated that serum sodium levels in the upper half of the “normal range” should be treated as a clinical risk factor that prompts recommendation for modification of water and salt intake.

## Results

### Water-restricted mice

#### Shortened life span.

**Figure 1:**
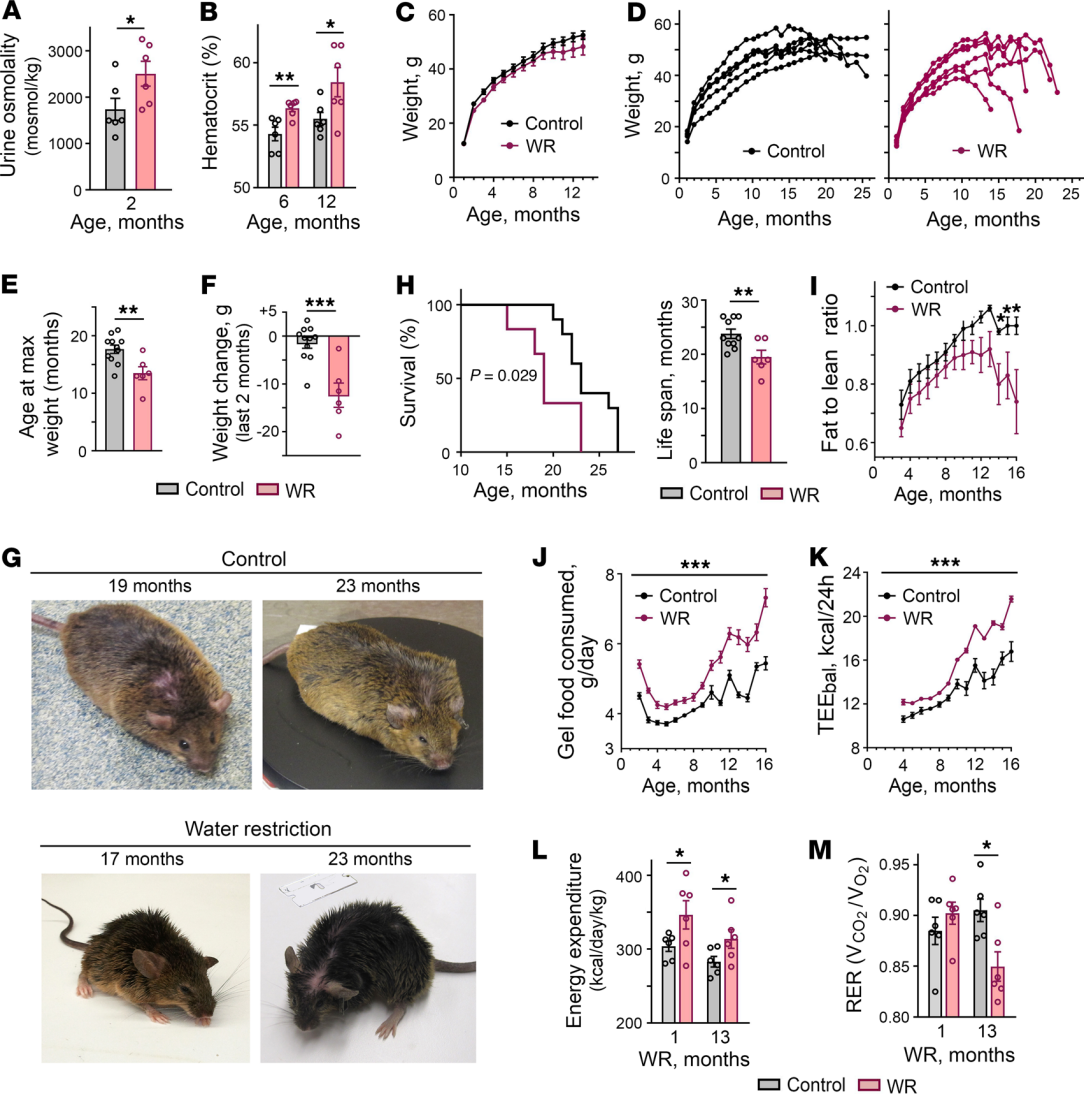
Mild lifelong water restriction shortens mouse lifespan accompanied by metabolic remodeling toward increased food intake and energy expenditure. Mice were water restricted throughout their entire lives by feeding them with gel food made from 30% of water and 70% of dry food without access to any additional water. (**A** and **B**) Water restriction results in chronic decrease in hydration level. (**A**) Water-restricted (WR) mice have elevated urine osmolality. (**B**) WR mice have elevated hematocrit. (**C**–**F**) Aging of WR mice is accompanied by a sharp decrease in weight during the last several weeks of life. (**C**) Control and WR mice grow at the same rate during the first year of life. (**D**) Representative growth curves showing sharp weight decrease of WR mice. (**E**) WR mice stop gaining weight at an earlier age. Data are plotted as age at maximum weight (mean ± SEM).**P < 0.01 by unpaired, 2-tailed *t* test. (**F**) Weight change during the last 2 months of life (mean ± SEM. ***P < 0.001 by unpaired, 2-tailed *t* test. (**G**) Representative pictures. WR mice are shown after they lost weight. They looked similar to control mice before the weight loss started. (**H**) WR mice have shortened life span. Left panel: the Kaplan-Meier survival curves (*P* = 0.029, log-ranked Kaplan-Meier survival analysis). Right panel: average life span (*t* test, unpaired 2-tailed, *P* = 0.039; Control: *n* = 11, WR: *n* = 6). (**I**) Attenuation of weight gain followed by weight loss is caused by decrease in body fat mass. Body composition analysis: fat-to-lean mass ratio (mean ± SEM, *P < 0.05 relative to water restriction by unpaired, 2-tailed *t* test). See Supplemental Figure 1 for fat and lean mass. (**J**–**M**) Water restriction increases energy expenditure. (**J**) WR mice consume more food. Daily food consumed per mouse is plotted as mean (of 30 days) ± SEM. *P < 0.001 relative to water restriction by unpaired, 2-tailed *t* test). (**K**) Estimation of energy expenditure by calculations of energy balance (TEE_bal_): caloric intake minus change in body energy stores. See Methods for details. WR mice have increased TEE_bal_ through whole period of water restriction. (**L** and **M**) Characterization of energy expenditure by measurement of gas exchange and heat production in calorimetric chambers. (**L**) Higher heat production in WR mice is consistent with increased energy expenditure detected by energy balance calculations shown on panel K (mean ± SEM, *n* = 6). *P < 0.05; **P < 0.01 by unpaired, 2-tailed *t* test). (**M**) Respiratory exchange rate (RER) decreases in WR mice after 13 months of water restriction consistent with higher proportion of metabolic utilization of lipids.

To find out what aspects of health are affected by chronic hypohydration, we exposed mice to mild lifelong water restriction starting at age 1 month by feeding them with gel food that contained 30% water and 70% dry food with no additional water provided. In our previous study, we demonstrated that this type of water restriction protocol increased serum sodium by 5 mmol/L and increased expression of NFAT5, a master regulator of hypertonic response, and its transcriptional target aldose reductase in many tissues including the liver, thymus, spleen, and kidney, indicating increased tonicity in extracellular fluids (10). Throughout their lifetime, the mice were observed by veterinarians for signs of health problems. We analyzed their food consumption, weight, and body composition and performed minimally invasive tests to assess their health status. The mice easily adapted to such water restriction and showed no visible distress. Despite the adaptation, the water-restricted (WR) mice had elevated urine osmolality (Figure 1A) and slightly elevated hematocrit levels (Figure 1B), confirming a chronic state of mild dehydration. No differences in growth rate and weight were noted during the first year of life (Figure 1C). After 1 year of life, the WR mice, in succession, began a slowdown in weight gain followed by a sharp decrease in weight during the last several weeks of life (Figure 1, D–G). The lifespan of WR mice was shortened by about 6 months (18%) compared with control mice that had free access to water (Figure 1H).

#### Metabolism remodeling.

Throughout the life of the mice, we performed a detailed analysis of body composition and food intake. Body composition did not differ from that of control mice until about 1 year of age, followed by a preferential decrease of body fat mass as the mice began to lose weight (Figure 1I and Supplemental Figure 1; supplemental material available online with this article; https://doi.org/10.1172/jci.insight.130949DS1). Analysis of the gel food intake revealed that WR mice consumed more food during the entire period of water restriction despite a same or lower weight (Figure 1J), indicating that their metabolism had changed toward higher energy expenditure. We confirmed stable elevation of the energy expenditure in WR mice by calculations of the energy balance (TEE_bal_) (Figure 1K) and by calorimetry (Figure 1, L and M) (24). The respiratory exchange rate (RER) was decreased in WR mice after 13 months of water restriction (Figure 1M), indicating a higher proportion of metabolic use of lipids and consistent with the decrease of fat mass that started after 1 year of life (Figure 1I and Supplemental Figure 1). These results indicated that water restriction caused stable metabolism adaptation characterized by increased energy expenditure.

**Figure 2:**
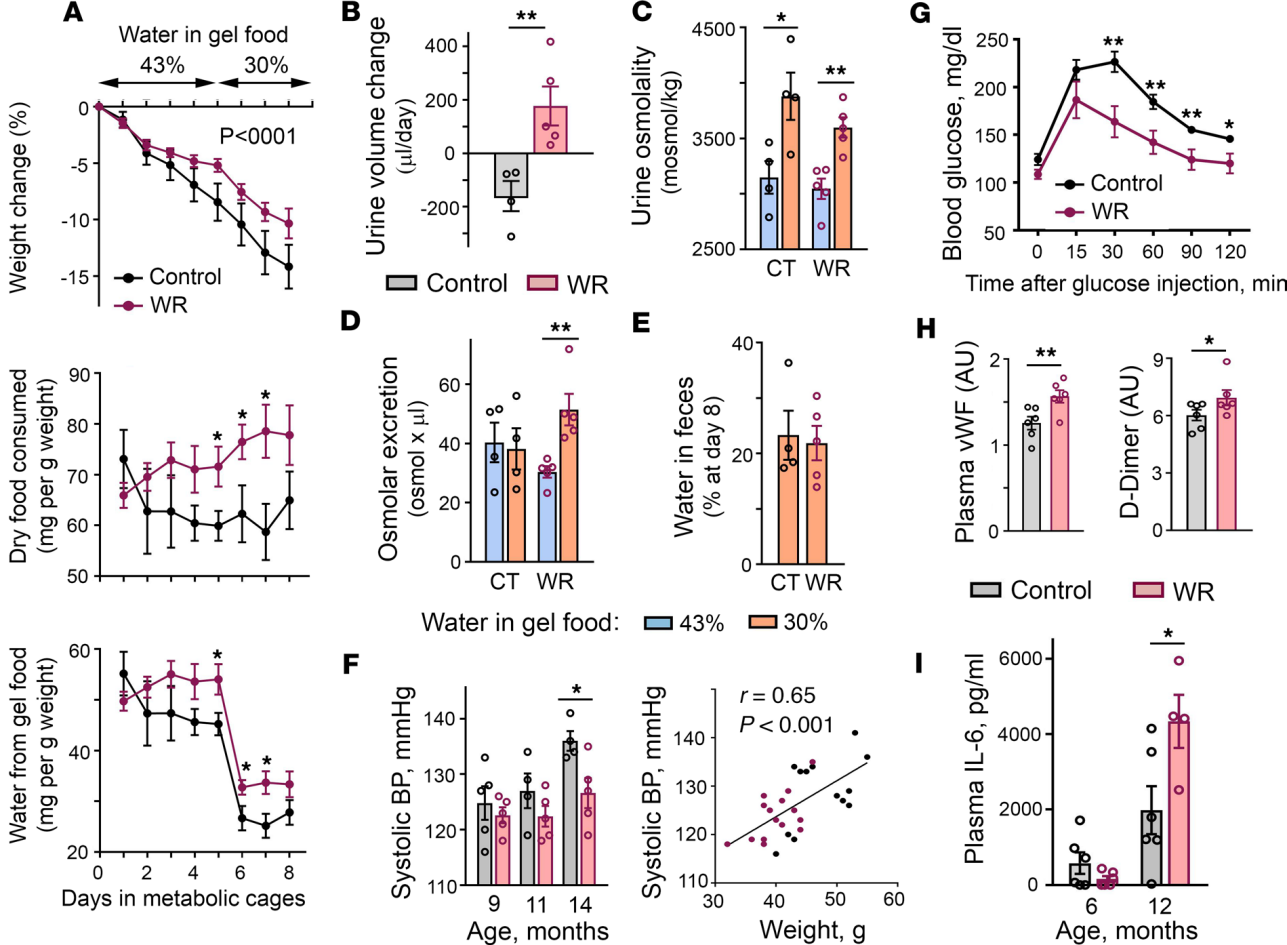
Effects of mild chronic water restriction on renal water conservation mechanisms and markers of inflammation and coagulation. (**A**–**E**) One-year water restriction does not worsen renal water conservation ability and remodels metabolism toward metabolic water formation. Mice were exposed to water restriction for 1 year and then provided with free access to water for 1 month. Efficiency of water balance regulation was assessed by exposing water-restricted (WR) mice and control mice (CT) to a short period of limited water availability performed in metabolic cages. Mice were given gel food containing 43% of water for 5 days, followed by a reduction to 30% water. No additional water was provided. (**A**) Time courses of food and water consumption and of weight changes. Top row: Both chronically WR mice and CT mice are losing weight with WR group at slightly lower rate. (*P* < 0.0001, test for the slopes difference). Middle and bottom rows: Upon reduction of water availability, CT mice decrease whereas WR mice increase food intake. (**B**) Despite decreased water consumption, WR mice increase urine volume indicating a fast switch of metabolism to metabolic water production. (**C**) Increased urine osmolality shows preserved kidney concentrating ability. (**D**) WR mice increase osmolar excretion consistent with metabolic water production from excess of consumed food. (**E**) Similar water content in feces indicates that this water preservation mechanism is not changed in WR mice. (**F**) Blood pressure (BP) measurements. Left panel: WR mice have lower BP (mean ± SEM). *P < 0.05 by unpaired, 2-tailed *t* test. Right panel: Analysis of correlation between BP and weight. All measurements for both groups and all time points are combined (*n* = 27). Significant correlation indicates that weight rather than water restriction determines BP variability (Pearson’s correlation coefficient = 0.65, *P* = 0.0002). (**G**) WR mice demonstrate faster glucose clearance in glucose tolerance test performed at age 16 months (mean ± SEM; Control: *n* = 5; WR: *n* = 4). **P < 0.01; *P < 0.05 by unpaired, 2-tailed *t* test. See Supplemental Figure 2 for mouse weights. (**H**) Increased levels of markers of inflammation and coagulation in chronically WR mice. Levels of vWF and D-Dimer are slightly elevated in WR mice at age 5 months. Quantification by densitometry (mean ± SEM). *P < 0.05; **P < 0.01 by unpaired, 2-tailed *t* test. See Supplemental Figure 3 for Western blot images. (**I**) Plasma IL-6 level increases faster with age in WR mice (mean ± SEM). *P < 0.05 by unpaired, 2-tailed *t* test.

Changes in the metabolism that chronic mild water restriction elicited in our laboratory mice (Figure 1, J–M) resembled the osmoregulatory adaptation mechanisms of desert animals. Desert animals can produce enough water from the oxidative metabolism of food to cover up to 90% of their water needs (25). In laboratory settings, acute water deprivation of the desert hopping mouse *Notomys alexis* induces appetite leading to sustained high food intake and remodels metabolism to enhance metabolic water production and stay in water balance (26). Therefore, in our next experiment, we tested whether chronic water restriction resulted in a similar adaptation in our mice. For this experiment, a separate group of mice were subjected to water restriction for 1 year and then were given free access to water for 1 month to provide them the opportunity to return to a state of euhydration. The mice were then exposed to acute water restriction for 8 days and their water conservation mechanisms were analyzed in metabolic cages (Figure 2, A–E). The mice subjected to chronic water restriction (chronically WR mice) showed a response that largely deviated from the response of control mice that had never been subjected to water restrictions. The normal response to acute water restriction or deprivation includes increased secretion of AVP in response to increasing plasma osmolality; in turn, this action leads to a decreased urine output because of increased water reabsorption by the kidney (27–29). Acute water restriction also normally leads to decreased food intake that is termed “dehydration anorexia” and serves to decrease the amount of solutes present within the body (27). Control mice demonstrated the classical response: as water availability decreased, they decreased food intake (Figure 2A, middle row), and excreted less urine (Figure 2B) that had higher osmolality (Figure 2C). Chronically WR mice however, similar to the desert mice, immediately increased food intake (Figure 2A, middle row). When the amount of water in gel food decreased from 43% to 30%, the mice excreted more urine (Figure 2B) despite decreased water intake (Figure 2A, bottom row), indicating that their metabolism switched toward metabolic water production from the consumed food. Part of this additionally produced water and additional solutes from excess food were excreted in the urine, leading to increased urine volume (Figure 2C) and increased osmolar excretion (Figure 2D). Although increased urine volume could potentially occur because of a decline in kidney function (29), the increased volume was not the result of impairment of urine concentrating ability because the urine osmolality increased normally (Figure 2C) and the mice had a lower rate of weight loss throughout the course of acute water restriction (Figure 2A, top row). Water content in feces was similar, indicating that this water conservation mechanism was not affected (Figure 2E). These results indicated that WR mice remodeled metabolism toward metabolic water formation that allowed them to respond efficiently to a water deficit and stay in water balance. Conversely, to achieve such efficiency the WR mice had to increase energy expenditure (Figure 1, J–M). This reaction is a risk factor for accelerated aging (18) and could contribute to a decreased life span (Figure 1H).

#### Inflammation, coagulation, and degenerative changes.

To further explore the possible origins of the decreased life span of WR mice, we analyzed other known morbidity and mortality risk factors. Elevated BP is a strong cardiovascular risk factor (30) that could contribute to the decreased lifespan of the WR mice. However, BP was lower in WR mice and was more strongly correlated with weight differences than with water intake in this model (Figure 2F). Increased blood glucose levels and glucose intolerance were additional risk factors and possible contributors to the premature death of the WR mice (31). However, at age 16 months the glucose level of the WR mice that had maintained their weight was similar to that of control mice and they performed even better in the glucose tolerance test (Figure 2G and Supplemental Figure 2).

Because previous studies have shown that short 9-day water restriction and elevated extracellular sodium in cell culture experiments elevate proinflammatory and prothrombotic mediators (10–13), we tested the levels of the markers of inflammation and coagulation in the chronically WR mice. Similar to mice subjected to short water restrictions (10), the blood levels of vWF and D-Dimer in the chronically WR mice were still slightly elevated after 5 months of the water restriction (Figure 2H), indicating that a low-grade prothrombotic state persisted throughout the entire period of the water restrictions. To test the level of inflammation, we measured the blood concentration of IL-6, the inflammatory marker that is elevated with age and most consistently associated with age-related chronic diseases, disability, and mortality (32). After 5 months of water restriction, the blood level of IL-6 was low and there was no difference between the control and WR mice (Figure 2I). Consistent with its age-dependence, the level of IL-6 increased in both groups by age 14 months, but to a larger degree in WR mice (Figure 2I), indicating that age-related proinflammatory changes were accelerated by chronic hypohydration. These results indicated that the WR mice had been in a state of chronic subclinical inflammation and coagulation that are well-recognized modifiers of the rate of aging (17, 20).

**Figure 3:**
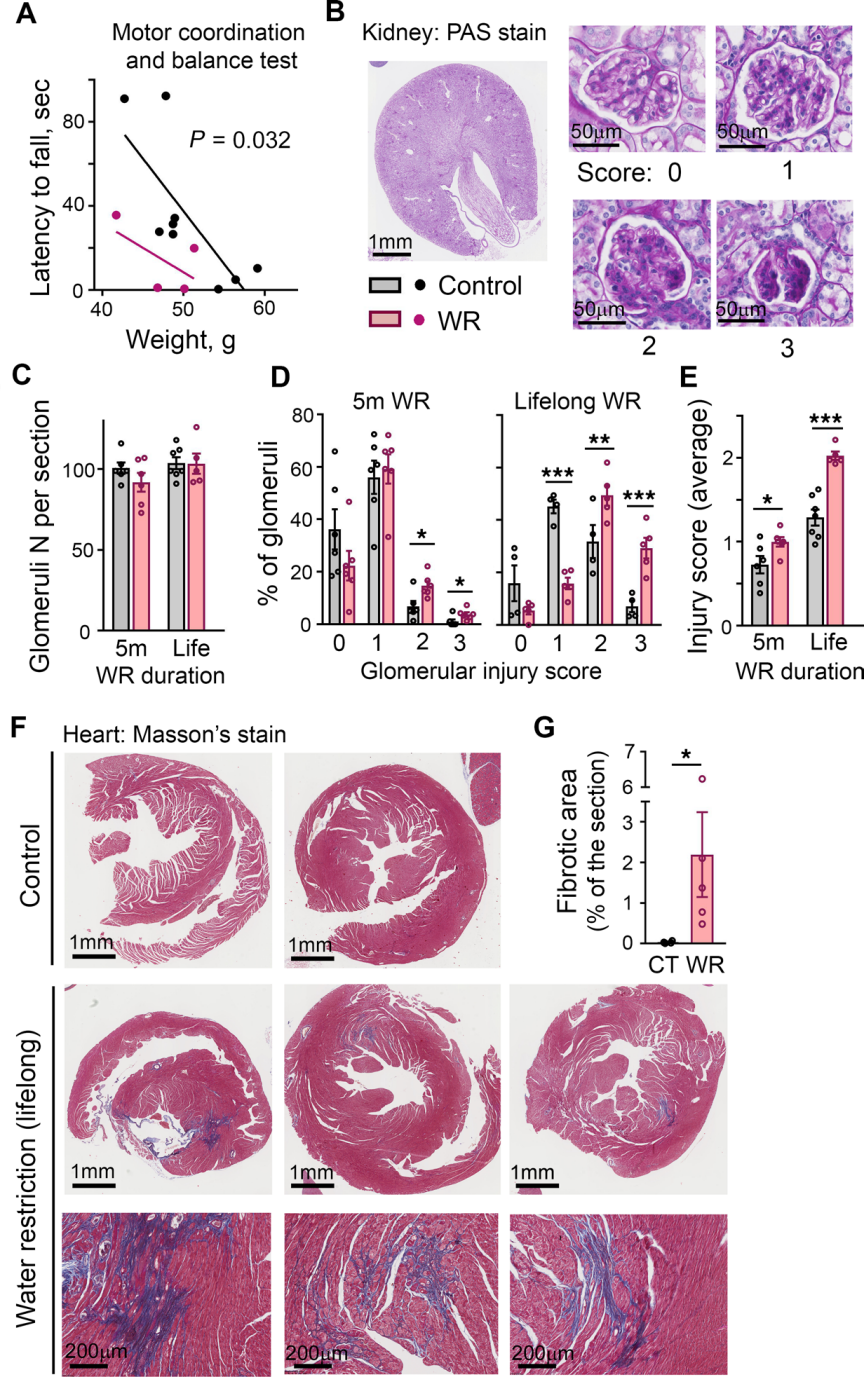
Accelerated impairment of neuromuscular coordination, accumulation of renal glomeruli injuries, and development of cardiac fibrosis in chronically water-restricted (WR) mice. (**A**) WR mice have impaired motor coordination assessed by Rota Rod test at age 14 months. Data are presented as latency to fall versus weight (*P* = 0.032, ANCOVA for differences between regression lines elevations). (**B**–**E**) Renal deterioration is accelerated in WR mice. (**B**) Representative images of periodic acid-Schiff–stained mid-kidney cross-section and examples of glomeruli for each scoring category used to quantify degree of glomerular injury, from 0 (no injury) to 3 (globally sclerotic glomeruli). The analysis is done after 5 months of water restriction and at the end of lifespan. (**C**) Number of glomeruli does not change throughout life both in control and WR mice. (**D**) Proportion of total glomeruli per injury score category. (**E**) Mean glomeruli injury score. Accumulation of glomerular injury is accelerated in WR mice (mean ± SEM). *P < 0.05; **P < 0.01; ***P < 0.001 by unpaired, 2-tailed *t* test. (**F** and **G**) Water restriction promotes cardiac fibrosis. (**F**) Images of Masson’s trichrome stain of the heart sections at the end of lifespan. Blue color identifies collagen fibers. Bottom panels: magnifications of fibrotic areas in the heart of WR mice. (**G**) Quantification of fibrotic areas as percent of total section areas (mean ± SEM). *P < 0.05 by unpaired, 2-tailed *t* test.

In summation, our analysis of the long-term effects of chronic hypohydration in a mouse model demonstrated that during the first 12–14 months of life, despite an absence of obvious clinical symptoms, the mice had been in a state of increased energy expenditure and low-grade inflammation and coagulation. These conditions are recognized as modifiers of the rate of aging and are stimulators and indicators of age-related degenerative decline (17–20). At age 14 months, WR mice demonstrated signs of faster decline of motor coordination as evidenced by their inferior performance on the Rota Rod test (Figure 3A). Examination of postmortem tissues revealed a higher degree of renal glomerular injury (Figure 3, C–E) and cardiac fibrosis (Figure 3, F and G) in the WR mice. More glomeruli injury was already detectable after 5 months of the water restriction (Figure 3, C–E).

### Hypohydration outcomes in humans

#### Overview of the analysis.

**Figure 4:**
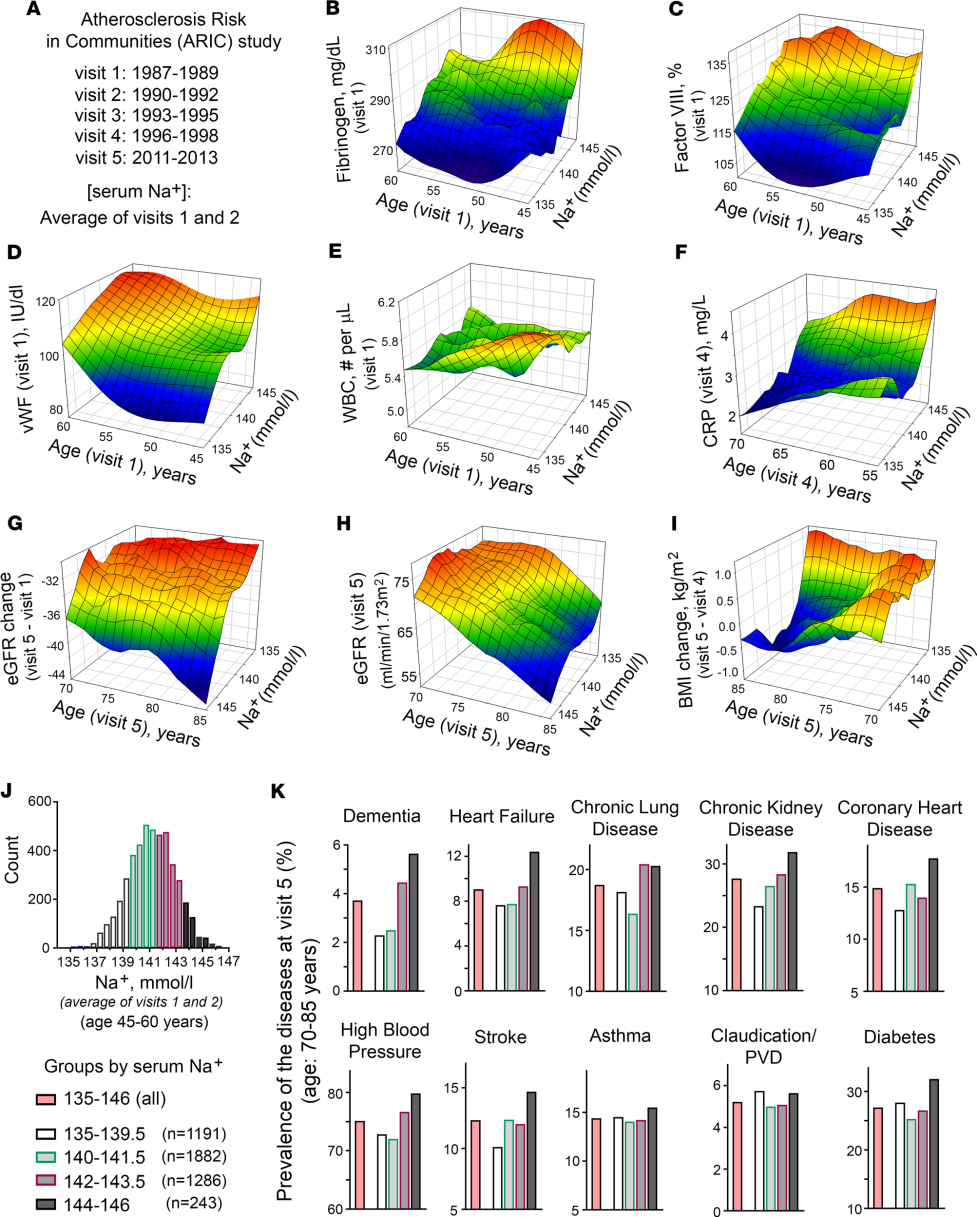
Hydration status assessed by serum sodium concentration at middle age is associated with markers of inflammation and coagulation and predicts development of age-related degenerative diseases 24 years later: Atherosclerosis Risk in Communities (ARIC) study. (**A**) Overview of ARIC study. ARIC is a prospective epidemiologic study that recruited 45- to 64-year-old participants (15,792 total) in 1987–1989 and followed them for 24 years. The follow-up included 4 additional visits and telephone interviews. Current analysis included participants who had all analyzed variables available, had average serum sodium concentration from visits 1 and 2 within reference range of 135–146 mmol/L and average glucose concentration at visits 1 and 2 lower than 126 mg/dL. In all, 4,602 participants remained for the analysis. (**B**–**F**) 3D mesh plots, visualizing continuous variables as functions of serum sodium concentration and age observed in the ARIC study participants. See Table 1 for results of formal linear regression analyses and Supplemental Figure 4 for distributions of the variables. Participants with higher serum sodium levels (**B** and **C**) had increased level of acute-phase proteins fibrinogen and factor VIII at visit 1, (**D**) had higher level of vWF at visit 1, (**E**) did not change white blood cell count (WBC), (**F**) had higher level of C-reactive protein (CRP) at visit 4, (**G** and **H**) showed faster decline in estimated glomerular filtration rate (eGFR) with age, and (**I**) lost weight during last 15 years of follow-up (between visits 5 and 4). (**J** and **K**) Prevalence of diseases in ARIC study participants at visit 5 depending on average serum sodium concentration measured at visits 1 and 2. (**J**) Distribution histogram of average serum sodium concentration on visits 1 and 2 in ARIC study participants included in the analysis. Participants are divided into 4 groups based on their serum sodium concentrations. (**K**) Prevalence of the diseases in the groups with different serum sodium concentrations. Higher sodium is associated with higher prevalence of many chronic diseases with highest prevalence in the 143–146 mmol/L group for all diseases except asthma and peripheral vascular disease (PVD) and with a sharp increase at 142 mmol/L for dementia, heart failure, and chronic lung diseases. See Table 1 and Supplemental Table 2 for results of formal logistic regression analyses.

Based on our mouse studies, we hypothesized that hypohydration in humans could induce a lifelong proinflammatory state and metabolism remodeling and also accelerate age-related degenerative changes. To test the health outcomes of different hydration levels in humans, we analyzed data from the ARIC study (33). ARIC is an ongoing population-based prospective cohort study in which 15,792 adults aged 45 to 64 years were enrolled from 1987 to 1989 (33). The participants were evaluated in person over 5 visits spanning 24 years until 2011 to 2013 (Figure 4A). We used serum sodium concentration as a measure of hydration status because in a healthy person with no major disease affecting the water/electrolyte balance, such as advanced-stage chronic kidney disease (CKD), diabetes mellitus, or HF, serum sodium concentration is a principal determinant of extracellular osmolality (water balance outcome) (34). In the ARIC study, the serum sodium concentration was measured during visits 1 and 2 and we used the average concentration from these 2 measurements. We performed the analysis under the assumption that these sodium measurements would give an estimate of lifelong hydration. We made this assumption based on a study that evaluated the clinical records of more than 150,000 people and revealed that serum sodium concentration had very little variability in individual persons over a 10-year period (22). In addition, our previous analysis showed that participants from the ARIC study had stable serum sodium concentrations within 2–3 mmol/L between visits 1 and 2 that took place 3 years apart (23). The reason for such stability is not clearly established and could be affected by lifestyle habits for water and salt intake and by genetic differences in water balance regulation (35–37). In any case, such stability indicates that hydration status is an individual characteristic that persists for long time spans and can therefore affect lifelong health outcomes. For the analysis, we included participants who had all analyzed variables available from visit 1 to visit 5. We excluded people who had a sodium concentration outside the reference range of 135–146 mmol/L and a plasma glucose level higher than 126 mg/dL. This restriction was instituted to decrease the number of people whose sodium concentration did not represent their true hydration level because it was shifted as a result of hyperglycemia or other major abnormalities of water-electrolyte homeostasis (38, 39). In all, 4,602 participants remained for the analysis.

#### Inflammation and coagulation markers.

To test the relationship of serum sodium concentration with the markers of inflammation and coagulation, we performed multiple linear regression analysis. The analysis revealed that at middle age, higher serum sodium concentration was positively associated cross-sectionally with increased levels of acute-phase proteins fibrinogen and factor VIII and with vWF (Table 1). Figure 4 shows 3D mesh graphs illustrating these relationships between serum sodium, age, and each of the markers. The graphs demonstrated that higher levels of serum sodium concentration at all ages corresponded to higher levels of fibrinogen (Figure 4B), factor VIII (Figure 4C), and vWF (Figure 4C). This increase was not related to an infection because there was no significant association of the serum sodium with WBC counts (Table 1 and Figure 4E). A higher sodium level at middle age was also positively associated longitudinally with elevated C-reactive protein (CRP) at follow-up visits 4 and 5 (Table 1 and Figure 4F). Previous analysis of ARIC participants also showed cross-sectional association of serum sodium concentration with BP and plasma lipids (23). Taken together the results indicated that participants whose serum sodium concentration was close to the upper end of normal reference range had higher levels of risk factors for age-related morbidity and mortality (17, 40, 41).

#### Degenerative diseases.

Although we detected statistically significant associations of serum sodium concentration with multiple risk factors (Table 1, Figure 4, C–F, and ref. 23) in ARIC study participants at middle age, it is worth noting that the increases in the levels and concentrations of risk factors associated with sodium variations within the normal range are very moderate: all levels stayed within their own normal range (Figure 4 and Supplemental Table 1) and would not normally trigger any clinical concern. Only the CRP at visit 4 was slightly elevated above normal range in people whose sodium concentration exceeded 143 mmol/L (Figure 4F and Supplemental Table 1). To determine whether such moderate elevations produce clinically relevant changes in the long run, we next tested whether higher levels of serum sodium predict faster development of age-related degenerative changes. We performed multiple logistic regression analysis of association between serum sodium at middle age with development of age-dependent diseases 24 years later at visit 5.

The association of serum sodium with the development and progression of CKD has been established in previous studies (42–44). In our analysis, higher serum sodium even within the normal range was associated with a larger decline of calculated eGFR between visits 1 and 5 spanning 24 years (Table 1 and Figure 4G), leading to a lower eGFR at visit 5 (Figure 4H) with a higher proportion of people that had developed CKD by visit 5 with an eGFR of less than 60 mL/minute/1.73 m^2^ (Figure 4K). In our mouse model, the drastic outcome of lifelong hypohydration was sharp weight loss during the last several weeks of life (Figure 1, D, F, and G). Similarly, in the ARIC study participants, serum sodium measured at visits 1 and 2 was associated with weight loss during the last 15 years of the study from visit 4 in 1996–1998 to visit 5 in 2011–2013 (Table 1 and Figure 4I). This result demonstrates that responses to chronic hypohydration are similar in a mouse model and humans.

In our mouse model, chronic hypohydration promoted degenerative changes leading to the accelerated development of renal glomerular injury (Figure 3, B–E), cardiac fibrosis (Figure 3, F and G), and the deterioration of motor coordination (Figure 3A). Similarly, multiple logistic regression analysis of ARIC study data revealed significant positive associations of the serum sodium at middle age with dementia, HF, CLD, high BP, and diabetes mellitus that have developed 24 years later by visit 5 (Table 1). Asthma, atrial fibrillation, peripheral vascular disease (PVD), coronary heart disease (CHD), and stroke did not show significant association in this type of analysis (Supplemental Table 2). As an alternative analysis, we divided the study participants into 4 groups based on their average serum sodium concentration from visits 1 and 2 (Figure 4J) and calculated the prevalence of diseases in these groups at visit 5 (Figure 4K). All diseases, except asthma, claudication, and PVD, had the highest prevalence in the 144–146 mmol/L group (Figure 4K). The most remarkable dependence on serum sodium concentration was seen for dementia: the prevalence of the disease increased 2 times in the 142–143.5 mmol/L group and almost 3 times in the 144–146 mmol/L group compared with people who had a serum sodium concentration of lower than 142 mmol/L. A similar trend with increase of the disease prevalence when average serum sodium exceeds 141.5 mmol/L was seen for persons with HF, CLD, and high BP. To exclude the possibility that observed associations of serum sodium at visit 1 with the diseases prevalence at ages 70–85 years are dominated by participants who already had the diseases at initial examination, we performed additional analysis in which we excluded 332 participants who at visit 1 already had a diagnosis of HF, CHD, myocardial infarction, and diabetes mellitus. Such exclusion did not change the outcome of the analysis (Supplemental Figure 5).

**Figure 5:**
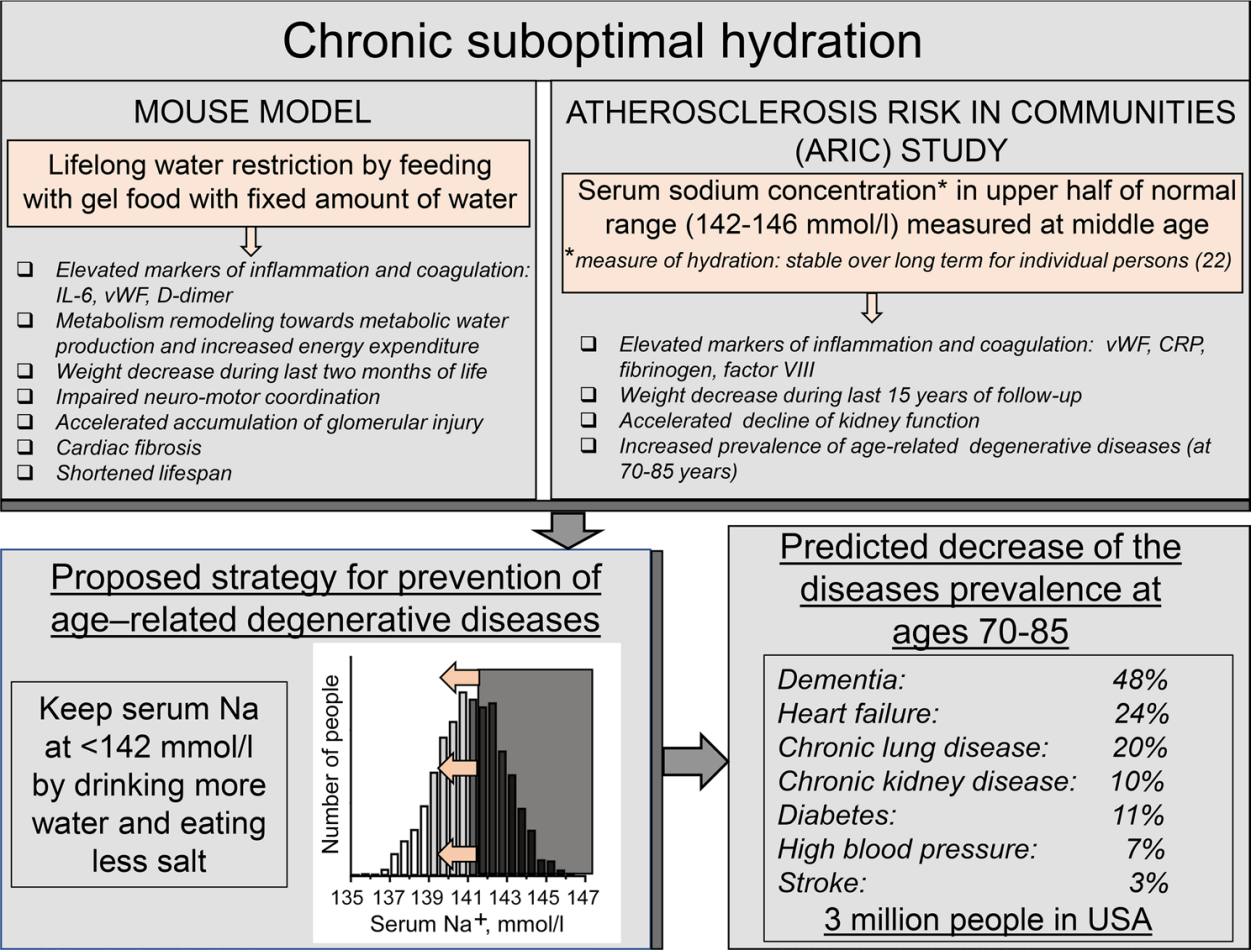
Study results overview, proposed strategy for decreasing risk of age-related degenerative diseases, and predicted implications for disease prevalence. Striking similarity between outcomes of lifelong water restriction in mouse model and of chronic hypohydration assessed by serum sodium concentration in humans suggest that chronic hypohydration promotes development of age-related degenerative diseases. Estimations based on the results from the Atherosclerosis Risk in Communities (ARIC) study predict that improving hydration and shifting serum sodium below 142 mmol/L has a potential to greatly reduce prevalence of the degenerative diseases.

By analyzing data from the ARIC study, we have demonstrated a strong association of serum sodium concentration measured at middle age with markers of coagulation and inflammation, and with the development of age-dependent degenerative diseases. The results of this analysis were remarkably similar to outcomes of chronic lifelong mild dehydration in a mouse model of water restriction (Figure 5). Difficult to control weight loss and increased inflammation, similar to factors we saw as a result of chronic hypohydration both in mice (Figure 1, D, F, and G) and in humans (Figure 4I), are factors commonly associated with many degenerative diseases, such as cognitive impairment and dementia (45–47), HF (48, 49), and CLD (50–52). Unexplained hypermetabolic state, similar to one elicited by hypohydration in our mouse model (Figure 1, J–M) is also seen in patients with these diseases (49, 52, 53). In the ARIC study itself, inflammatory markers measured at middle age are associated with increased odds of cognitive decline (47) and frailty (54). The similarity between the direct effects of hypohydration in a mouse model and associations of the diseases with elevated midlife serum sodium concentration in humans potentially suggest that lifelong hydration status is a causative determinant for the development of the age-related degenerative diseases.

## Discussion

The proinflammatory, procoagulation, and metabolic effects of subclinical chronic hypohydration revealed in our study are well-recognized modifiers of the rate of aging (17–19). It is well established that aging is accompanied by the development of a proinflammatory status termed “inflammageing” (17), as well as procoagulant and prothrombotic changes (20). Data from clinical studies indicate that elevated metabolic rate is also a morbidity and mortality risk factor (55–57). However, it is still debated whether inflammation and increased coagulation contribute causally to age-related morbidities or whether they are non-causal markers of aging itself. One of the possible inducers of the prothrombotic and proinflammatory effects of chronic hypohydration can be higher activity of the RAAS that is a well-established inducer of inflammation, coagulation, and oxidative damages (58–60) and would be expected to be activated in people whose serum osmolality exceeds the osmotic “set-point” of 280–290 mosmol/kg (4, 6, 8, 9). In addition, accumulating information suggests that elevated extracellular sodium can also directly affect these proaging responses. Thus, elevated extracellular sodium in cell culture stimulates the expression of proinflammatory and prothrombotic mediators, such as vWF, VCAM-1, E-selectin, and MCP-1 (10–12), reduces nitric oxide release (13), damages endothelial glycocalyx (13, 15), and affects lipids metabolism in cultured adipocytes (23).

An analysis of metabolic rates was not performed in the ARIC study. Therefore, we could not confirm its increase in relation to serum sodium, as it was found in the mouse model in relation to chronic hypohydration (Figure 1, K–M). However, data from other clinical studies of shorter durations indicate that an elevated metabolic rate is a morbidity and mortality risk factor in humans (55–57). Thus, in the Baltimore Longitudinal Study of Aging, a higher resting metabolic rate was associated with disease burden cross-sectionally (55) and predicted greater multimorbidity 4 years later (56). In a recent interventional study, a well-controlled calorie restriction diet over 2 years resulted in a metabolic adaptation in which energy expenditure decreased beyond that expected by weight loss and was accompanied by reduced urinary markers of ROS production (21). The study provided support in humans to the long-debated theory of the proaging effects of oxidative damages caused by ROS elevation induced by high metabolic rates (18, 61). Similar mechanisms could contribute to accelerated degenerative changes triggered by hypohydration in our present study in which increased demand for metabolic water production led to increased food intake and energy expenditure (Figure 1, K–M).

### Perspective: estimation of benefits from improved hydration.

The results presented in our current study provided evidence for a causative association of chronic subclinical hypohydration with the accelerated development of age-dependent degenerative diseases and their risk factors, such as increased energy expenditure and low-grade inflammatory and procoagulative states. These findings allowed proposing new preventive measures for extending a healthy lifespan based on maintaining optimal hydration status throughout the lifetime (Figure 5). Although the general importance of adequate hydration is generally recognized (2), systematic experimental animal studies of the long-term effects of suboptimal hydration, as well as long-term clinical studies, are lacking (5). Evaluations of average liquid consumption show failure to meet existing recommended dietary reference intake values, both in children and adults (62, 63). It is also recognized that establishing recommendations for water requirements that meet the needs of all persons is impossible because individual fluid needs differ because of variations of factors that influence water loss and solute balance, such as activity, nutrition, environment, and disease (64–66).

Our analysis of the ARIC study identified a clear threshold of 141.5 mmol/L for serum sodium concentration above which the risk of the development of chronic age-related diseases greatly increases (Figure 4K). This threshold could be easily used as a reference level for the development of individualized strategies for modifying water and salt intake aiming to slow down age-related degenerative changes (Figure 5). More studies will be needed, however, to determine how effective this strategy can be and how many people would be able to shift their sodium level by simple modifications of water and salt intake. Existence of individualized osmotic “set-points” for activation of AVP-mediated water preservation mechanisms (6, 9) could create challenges for serum sodium and osmolality decrease by simple modifications of water and salt intake for people whose higher sodium levels are the result of their higher osmotic “set-point” as opposed to habitual low water and high salt intakes.

To estimate the potential impact of shifting serum sodium concentration below 142 mmol/L by improving hydration on the diseases burden, we calculated the predicted decrease in the prevalence of diseases with the theoretical assumption that all people with average serum sodium concentration higher than 141.5 mmol/L would move to the 140–141.5 mmol/L group (Figure 4, J and K, and Supplemental Table 3). These calculations predicted that the prevalence of dementia in people aged 70–85 years would decrease by 48%, HF by 24%, CLD by 20%, CKD by 10%, diabetes mellitus by 11%, high BP by 7%, and stroke by 3.1% (Supplemental Table 3 and Figure 5). To estimate how many people would not develop these diseases as a result of such a preventive strategy, we extrapolated the results from the ARIC study analysis to the whole population of the United States in which about 27,560,000 people aged 70–85 years reside (US Census Bureau, 2015). The calculations predicted that there would be about 342,000 fewer people with dementia, 353,000 fewer with HF, 597,000 fewer with CLD, 422,000 fewer with CKD, 442,000 fewer with diabetes mellitus, 822,000 fewer with high BP, and 59,000 fewer with stroke, in total decreasing the number of people aged 70–85 years with these diseases by 3,000,000 in the United States alone (Supplemental Table 3 and Figure 5).

## Methods

### Mice

Male 129S6 traditional inbred mice (129S6, model no. 129SVE-M, Taconic Farms, Inc) were purchased at the age of 4 weeks. All mouse studies were carried out in strict accordance with the recommendations in the *Guide for the Care and Use of Laboratory Animals* of the NIH (National Academies Press, 2011). The mice were housed in an Association for Assessment and Accreditation of Laboratory Animal Care-accredited facility of the National Heart Lung and Blood Institute. At the end of the experiments, mice were euthanized by cervical dislocation.

### Water restriction

The mice were water restricted for their entire life starting from the age of 1 month. All mice received gelled food containing 30% water and 70% dry food (1.7 mL of deionized water + 4 g of balanced purified rodent diet, AIN-76A, Research Diets, + 57 mg of agar per 5.7 g of the food). Food was provided in excess in individual cups, thus the mice ate what they wanted. The control group had free access to water. WR mice did not get any additional water. Three mice were housed per cage. The cups were replaced every day and the amount of food eaten by mice in each cage was measured by weighing the cups and averaged per mouse per day. The mice were housed with 14-hour light/10-hour dark cycle at 20°C–24°C in polycarbonate cages with a wire lid holding chow and water bottle and paper-based bedding. All acute tests and measurements were performed at daytime between 8 am and 5 pm.

### Survival analysis

Time of death was recorded using the date mice were found dead, or the time when the mice were determined to be moribund and/or displaying such severe discomfort that veterinary technicians recommended euthanasia. The mice were euthanized by cervical dislocation.

### Body composition

Body fat and lean tissue were measured using Mini-Spec Body Composition Analyzer (Bruker). Unanesthetized mice were placed in a clear plastic tube and gently restrained at the end of the tube by using a plunger with air holes to minimize movement. The tube was then inserted into the mini-spec port to a premeasured depth and the measurements were collected.

### Estimation of energy expenditure using energy balance method (EBM)

In EBM, energy expenditure (TEE_bal_) is determined by calculating the difference between metabolizable energy intake (MEI = energy in consumed food) and changes in somatic energy stores. The calculations were performed as previously described (24):

### TEE_bal_ = MEI – (Δ somatic fat energy + Δ somatic lean energy)

MEI was calculated as grams of ingested food per day multiplied by energy content in 1 g of the mice diet (3.9 kcal/g: AIN-76A, no. D10001, Research Diets). We estimated changes in somatic energy stores from fat and lean mass measurements described in “Body composition” section. Caloric equivalents were assigned as 9.4 kcal per gram of fat mass and 1.0 kcal per gram of lean mass as previously described (24).

### Estimation of energy expenditure using calorimetry

Indirect calorimetry was performed using Oxymax-CLAMS system (Columbus Instruments). The mice were singly housed in the calorimetry chambers for 5 days. The rate of the heat production was recorded for each mouse every 30 minutes as kcal/hour. To minimize the confounding effect of stress because of changes in the environment, only measurement from day 4 and 5 were used in the final analysis.

### Analysis of urinary concentrating ability and response to acute water restriction

The test was performed when mice were age 14 months. There were 2 groups of mice: (a) Control mice were fed gel food containing 30% of water and had free access to water throughout entire life; (b) Chronically WR mice were fed with the same gel food and did not have additional water until 13 months of age and then were given free access to water for 1 month. To test response to acute water restriction, the mice were placed in metabolic cages (Hatteras Instruments) with controlled temperature and light (12-hour light and dark cycles). All mice received gelled food containing 43% water (3 mL of water + 4 g of balanced purified rodent diet (AIN-76A, Research Diets) + 70 mg of agar) per 7 g of the food. Food was provided in excess in individual cups, thus the mice ate what they wanted. After 5 days, the amount of water in the gel food was decreased to 33% (2 mL of water + 4 g of the rodent diet powder + 60 mg of agar) for 3 more days. Body weight, food consumption, urine volume, and urine osmolality were measured every 24 hours. Urine was collected under mineral oil in preweighed collection vials that were replaced every 24 hours. The volume was measured gravimetrically, assuming a density of 1. Gel food was supplied in preweighed plastic cups to facilitate measurement of food consumption. Urine osmolality was measured with a Fiske Model 210 Freezing-Point Micro-Osmometer (Fiske Associates). Water content of feces was measured by collecting feces in preweighed tubes and weighing the tubes before and after drying in a chemical hood for 3 days.

### BP measurements by tail-cuff method

Systolic BP was measured noninvasively in conscious mice by the tail-cuff method using MC4000 Blood Pressure Analysis System (Hatteras Instruments Inc). Mice were placed in restrainers in which the tail was positioned inside an inflatable cuff. Before testing, all mice were trained to become accustomed to short-term restraint. For mice acclimation to the procedure, BP was recorded for 2 days with 10–15 measurements per session. The test measurements were performed for 4 consecutive days with 10–15 measurements for each test session. For final analysis, the readings from the 4 test sessions were averaged for each mouse.

### Intraperitoneal glucose tolerance test (GTT)

The test was performed at age 16 months. To exclude factors related to poor health on the results, the test was performed only on mice that were not losing weight at the time. See Supplemental Figure 2 for information about weight of mice used in this experiment. The mice fasted for 6 hours before the test. A drop of blood for the glucose measurements was collected from the tail by cutting the tail gently with a sterile scalpel blade close to tail tip. I.P. injection of 100 mg glucose per mouse in the form of 10% glucose solution in normal saline (2 mg/g mouse weight on average) was done at 0 time and then blood glucose was measured 15, 30, 60, 90, and 120 minutes after injection.

### Mouse blood collection and plasma isolation

Blood was collected from the tails of the mice. The mice were placed under a heat lamp for 3–5 minutes to warm mice and dilate blood vessels. After warming, the mice were placed in a restraining device from which their tails protruded. A small nick was made in the tail with a sterile scalpel blade. Four drops of the blood were collected into a tube containing 15 μL of sodium citrate (3.2%). Pressure was applied to the nick for 15–30 seconds to stop the flow of blood. Tubes were centrifuged at 1000 *g* for 10 minutes. Plasma was transferred to new tubes, then centrifuged for 10 more minutes at 10,000 g to remove platelets. The platelet-free plasma was stored at −80°C. Collection tubes were weighed before and after the blood collection, and after the plasma was transferred to determine volumes of the collected plasma and of blood cells. Coefficient of dilution by sodium citrate was calculated as V_plasma_/(V_plasma_ – V_sodium_ _citrate_) to correct the concentrations of the plasma analytes after measurements. Hematocrit was estimated as V_cells_/(V_plasma_ + V_cells_ – V_sodium_ _citrate_).

### Measurement of vWF and D-Dimer in the mouse plasma

vWF and D-Dimer in the plasma were measured by Western blot at age 5 months. Equal volumes of the plasma were loaded on the gel. Before loading, 3× Laemmli Sample buffer supplemented with dithiothreitol (no. 7722, Cell Signaling) was added to the samples and the mixtures were boiled for 5 minutes. Immunoblots used primary antibodies against vWF (no. A0082, Dako) and D-Dimer (no. bs-3514R, Bioss) and secondary antibodies labeled with Alexa Fluor 680 nm Dye (Invitrogen). The immunoblots were scanned and integral fluorescence from each band was measured using Odyssey Infrared Imaging System (Li-COR Biosciences). See complete unedited blots in the supplemental material. For final quantification, we corrected the measurements for plasma dilution with sodium citrate by multiplying by the coefficient of dilution calculated as described in the *Mouse blood collection and plasma isolation* section.

### Rotarod testing

Rota Rod Rotamex 5 (Columbus Instruments) was used to assess motor coordination, balance, and equilibrium. The mice that were already losing weight were excluded from the analysis. One day before testing, training sessions ware performed. During the training period, each mouse was first placed on the stationary rotarod for 3 trials with 10 minutes rest, followed by 3 trials on the rotarod at a constant speed (4 rpm) for a maximum of 60 seconds with 1 hour of rest. The next day, mice received 5 test trials in which the rotarod accelerated gradually from 0 to 20 rpm and the latencies for the mice to fall from the rod were recorded and used in analysis. Mean latency to fall from the 5 trials was calculated for each mouse. Because Rota Rod performance is dependent on weight, the statistical difference between groups was calculated using ANCOVA analysis with weight as covariate.

### Statistical analysis of the mouse data

Statistical analyses were done using GraphPad Prism 7 and SigmaPlot 13.0 software. All data were tested for normality before the analysis. If the normality test passed, comparisons were performed by the 2-tailed unpaired Student’s *t* test. If not, the nonparametric Mann-Whitney test was used. A *P* value of less than 0.05 was considered significant.

### Mouse study approval

The mouse protocol was approved by the Animal Care and Use Committee of the National Heart, Lung, and Blood Institute (protocol number: H-0130).

### Analysis of data from the Atherosclerosis Risk in Communities (ARIC) study

#### Data set.

We used data from the ARIC study. The data were obtained from the NHLBI Biologic Specimen and Data Repository Information Coordinating Center (BioLINCC). The data sets were redacted to remove personal identifiers to conform to the individual informed consent restrictions.

#### Study population.

ARIC is an ongoing population-based prospective cohort study in which 15,792 45- to 64-year-old black and white men and women were enrolled from 4 communities in the United States in 1987–1989. A detailed study design description has been published (33). Each community cohort was selected in the ARIC study by probability sampling to ensure that all individuals in an eligible age group had equal chances of being selected. Three subsequent visits were conducted at approximately 3-year intervals (visit 2 in 1990–1992; visit 3 in 1993–1995; visit 4 in 1996–1998) followed by visit 5 in 2011–2013 (Figure 4A). Participants have been contacted annually since baseline to obtain information about hospitalizations and for additional data collection. The ARIC study protocol was approved by the institutional review board of each participating center and informed consent was obtained from participants at each study visit.

#### Variables.

For our analyses, we used the following variables from the data collected and curated by the investigators of the ARIC study and obtained from BioLINCC data repository: serum sodium (visits 1 and 2), plasma glucose (visit 1), vWF (visit 1), fibrinogen (visit 1), factor VIII (visit 1), WBC count (visit 1), CRP (visits 4 and 5), and eGFR (visits 1 and 5). To analyze the development of the age-related diseases in ARIC study participants, we used variables from the ARIC study data indicating the presence or absence of the diseases by the time of visit 5. The following numeric indicator variables for the diseases were used: dementia, DEMDXL3_51, includes diagnosis during evaluation at visit 5 or telephone interview for cognitive status or informant interview, hospital discharge code, or death certificate code for dementia; HF, PREVSELFREPHF51, self-report of physician-diagnosed disease that was not contradicted by a later HF survey conducted by a physician; CLD, INCSELFREPLUNG5, self-report of physician-diagnosed disease; high BP, INCSELFREPHBP5, self-report of physician-diagnosed disease; diabetes mellitus, NCSELFREPDM51, self-report of physician-diagnosed disease; PVD or claudication, INCSELFREPCLD51, self-report of physician-diagnosed disease; asthma, NCSELFREPAST5, self-report of physician-diagnosed disease; atrial fibrillation, NCSELFREPAF51, self-report of physician-diagnosed disease; stroke, INCSELFREPSTK51, self-report of physician-diagnosed disease; CHD, PRVCHD51, diagnosis prior to visit 5. Detailed information about the analytes measurements in blood samples and about collection of information for numerical variables and their derivations for final databases is available at ARIC study website (https://www2.cscc.unc.edu/aric/cohort-manuals).

#### Exclusions.

Only participants who had all analyzed variables available from visit 1 to 5 were included in the analysis. For main analysis, we excluded people who had average sodium concentration from visits 1 and 2 outside the reference range of 135–146 mmol/L and plasma glucose level at visit 1 higher than 126 mg/dL. In all, 4,602 participants remained for the analysis. For additional analysis of diseases development in people free from the diagnosed diseases at visit 1, we excluded participants who already had by visit 1 prevalent HF (PREVHF01); CHD (PRVCHD05); history of myocardial infarction (HXOFMI02), and diabetes mellitus (DIABTS02). For this analysis, 4,270 participants remained (Supplemental Figure 5).

#### Statistical analysis of ARIC data.

Multiple linear regression analysis was used to assess associations of serum sodium with continuous variables: fibrinogen, factor VIII, vWF, WBC count, CRP, eGFR, and BMI change. Logarithmic transformation of CRP was performed to improve the normality of its distribution (Supplemental Figure 4). Multiple logistic regression analysis was used to asses associations of serum sodium with numeric variables describing occurrence of diseases. Serum sodium level was treated as the predictor variable. Age was included in all regression models as the second independent variable because it is a known strong predictor for most variables analyzed here. The analyses were performed using SAS (SAS Institute Inc) and SigmaPlot (Systat Software). The 3D mesh graphs shown on Figure 4 were constructed using SigmaPlot software with LOESS smoothing and rejection of outliers.

To calculate prevalence of the diseases at visit 5 depending on average sodium concentration at visits 1 and 2, the participants were divided into 4 groups based on their sodium level: 135–139.5 mmol/L (*n* = 1191); 140–141.5 mmol/L (*n* = 1882); 142–143.5 mmol/L (*n* = 1286); and 144–146 mmol/L (*n* = 243). Prevalence of the diseases at visit 5 was calculated as the percent of people with the disease in each of the 4 sodium groups.

#### Human study approval.

Transfer of ARIC data sets from NHLBI Biologic Specimen and Data Repository Information Coordinating Center (BioLINCC) repository was approved by the NIH Office of Human Subjects Research (OHSR) and was excluded from institutional review board review as Not Human Subjects Research, based on the interpretation of 45 CRF 46 under “Research Involving Coded Private Information or Biological Specimens” and Guidance on Engagement of Institutions in Human Subjects Research (October 16, 2008).

## Author contributions

MDA and DAS designed and performed mouse phenotyping experiments. MBB and MB secured funding and provided expertise and feedback. NID conceived the study, designed and performed mouse experiments and data analysis, analyzed data from the ARIC study, and wrote the manuscript.

## Supplementary Material

Supplemental data

## Figures and Tables

**Table 1:**
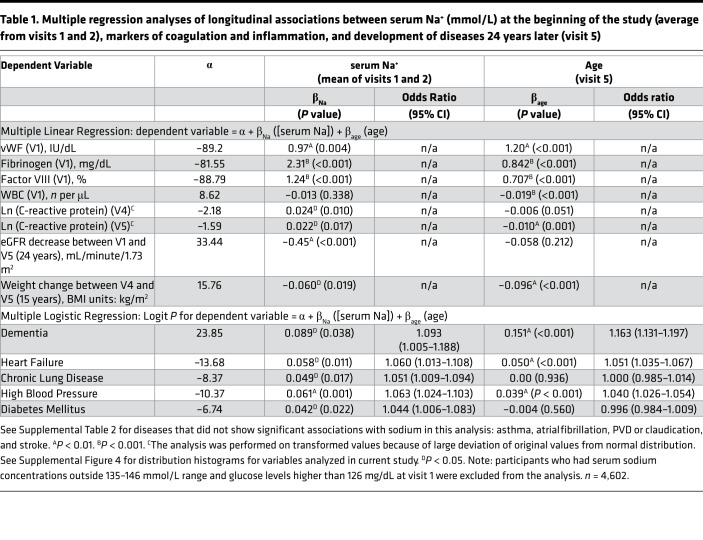
Multiple regression analyses of longitudinal associations between serum Na^+^ (mmol/L) at the beginning of the study (average from visits 1 and 2), markers of coagulation and inflammation, and development of diseases 24 years later (visit 5)

## Version 1 09/05/2019

Electronic publication
