# Supplementary material for: Suboptimal hydration remodels metabolism, promotes degenerative diseases, and shortens life
Source: JCI Insight. 2019 Sep 5;4(17):e130949. doi: 10.1172/jci.insight.130949 (PMC6777918; doi:10.1172/jci.insight.130949)
Supplement: Supplemental data [file jciinsight-4-130949-s154.pdf]

# Supplemental Figures and Tables

**Supplemental Figure 1 (related to Figure 1I). Body composition analysis: (A)** Fat mass; **(B)** lean mass (mean ± SEM,*P<0.05 relative to WR, unpaired two-tailed t test). Attenuation of weight gain followed by weight loss is caused by a decrease in body fat mass.

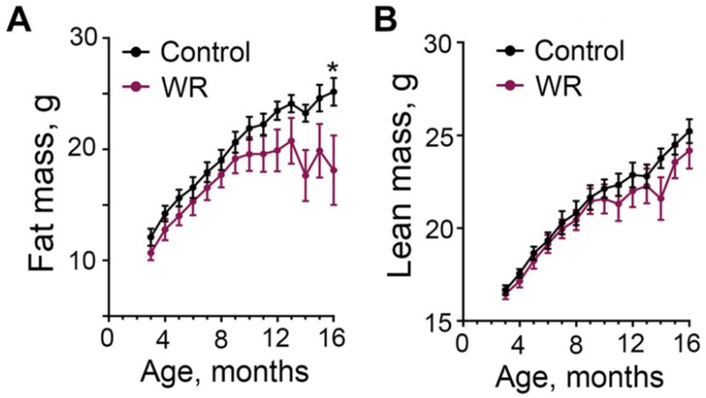

**Supplemental Figure 2 (related to Figure 2H). Weights of mice used for glucose tolerance test.** The test was performed at 16 months of age. For this test, we used mice that were not yet losing weight.

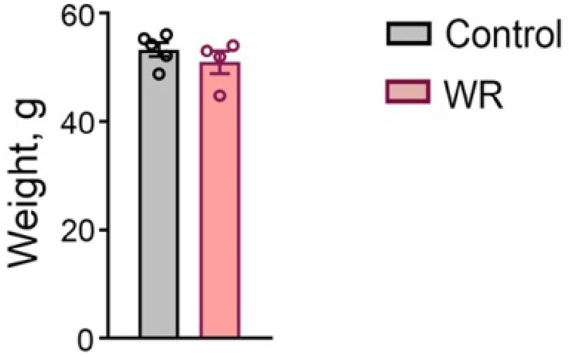

**Supplemental Figure 3 (related to Figure 2I). Western blot image used for quantification of vWF and D-Dimer in the mouse plasma.** Blood was collected from the mouse tail in 15ul of 3.2% sodium citrate. Equal volumes of plasma were loaded on the gel. The integral intensity of the bands was measured by densitometry and correction for the sodium citrate dilution was done for the final quantification shown on Figure 2I. See methods for more details.

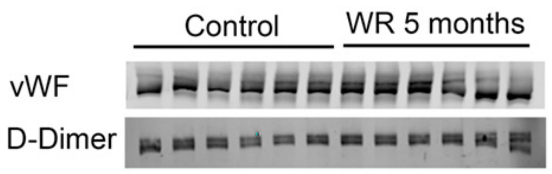

**Supplemental Figure 4. Distributions of the variables used in the Atherosclerosis Risk in Communities (ARIC) study analysis.** Out of 15,792 ARIC study participants enrolled from 1987 to 1989 only those who survived until visit 5 that took place from 2011 to 2013 were included in analysis. We excluded people who had average serum sodium concentration measured at visits 1 and 2 outside reference range of 135-146mmol/l and plasma glucose level at visit 1 higher than 126 mg/dL. These graphs show the distributions for the continuous variables for 4,602 participants remained for the analysis.

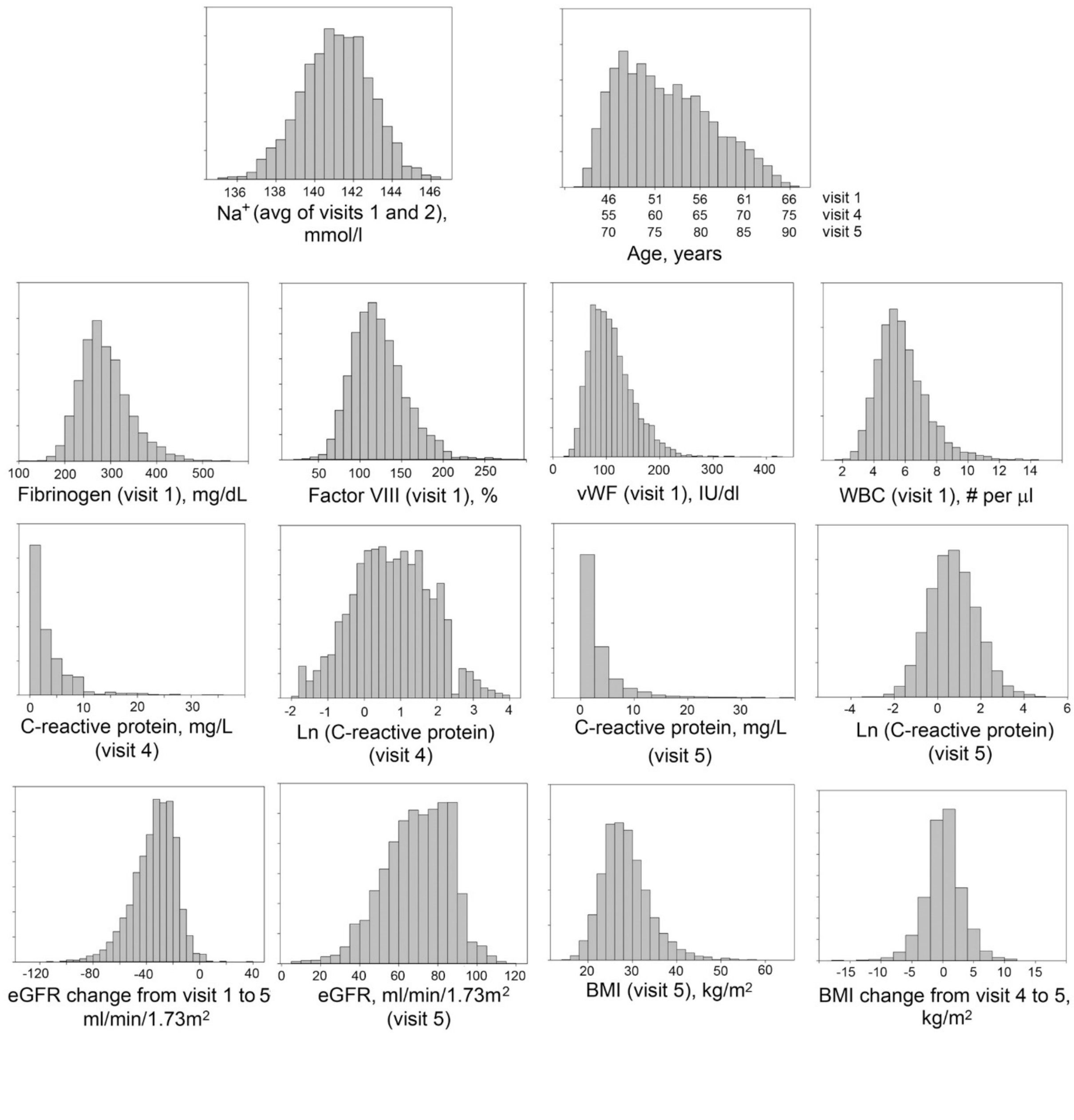

**Supplemental Figure 5 (related to Figure 4). Prevalence of diseases in Atherosclerosis Risk in Communities (ARIC) study participants at visit 5 depending on average serum sodium concentration measured at visits 1 and 2.** As compared to analysis presented on Figure 4, current analysis, in addition to excluding people with serum sodium outside 135-146 mmol/L range and blood glucose >126 mg/dL, also excludes participants who at visit 1 already had a diagnosis of *Heart Failure* (PREVHF01); *Coronary Heart Disease* (PRVCHD05); *Myocardial Infarction* (HXOFMI02) and *Diabetes* (DIABTS02). 4, 270 out of 4,602 participants remained for this analysis. **(A)** Distribution histogram of average serum sodium concentration on visits 1 and 2 in ARIC study participants included in the analysis. Participants are divided into four groups based on their serum sodium concentrations. **(B)** Prevalence of the diseases at visit 5 in the groups with different serum sodium concentrations at visits 1 and 2. Higher sodium is associated with higher prevalence of many chronic diseases with highest prevalence in the 143-146 mmol/l groups for all diseases except asthma and peripheral vascular disease (PVD) and with a sharp increase at 142 mmol/l for dementia, heart failure, and chronic lung diseases. <u>Conclusion:</u> *hydration status assessed by serum sodium concentration at middle age predicts development of age-related degenerative diseases 25 years later in people free from major diseases at initial examination.*

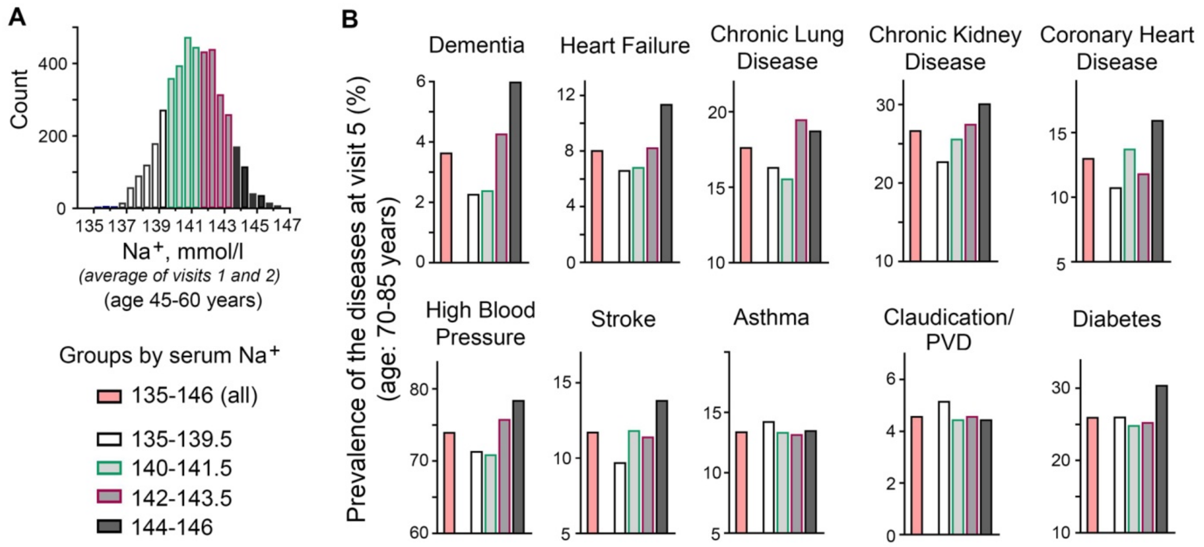

**Supplemental Table 1. Normal reference ranges for blood analytes analyzed in present study.**

| Variable | Reference range |
|---|---|
| Sodium, *mmol/l* | 135-145 |
| vWF, *IU/dL* | 50-200 |
| Fibrinogen, *mg/dL* | 150-400 |
| Factor VIII, % | 50-150 |
| CRP, *mg/L* | <3.0 |
| WBC, *N per µl* | 4-11 |

**Supplemental Table 2. Multiple regression analysis of longitudinal association between serum $Na^+$ (mmol/l) at the beginning of the study (average from visits 1 and 2) and development of chronic age-dependent diseases 25 years later (visit 5) (Atherosclerosis Risk in Communities (ARIC) study).** See also Table 1 for the diseases that showed significant association with sodium in this analysis.

| **Dependent variable** | **α** | **serum $Na^+$** (mean of visits 1 and 2) | | **Age** (visit 5) | |
|---|---|---|---|---|---|
| | | **$\beta_{Na}$ (P value)** | **Odds ratio (95% CI)** | **$\beta_{age}$ (P value)** | **Odds ratio (95% CI)** |
| *Multiple Logistic regression: Logit P for dependent variable = α + $\beta_{Na}$ ([serum Na]) + $\beta_{age}$ (age)* | | | | | |
| Asthma | -4.72 | 0.036 (0.119) | 1.036 (0.991-1.084) | -0.03*** (<0.001) | 0.973 (0.957-0.988) |
| Atrial Fibrillation | -9.31 | 0.029 (0.125) | 1.030 (0.992-1.069) | 0.054*** (<0.001) | 1.055 (1.042-1.069) |
| PVD or Claudication | -8.90 | 0.019 (0.606) | 1.019 (0.949-1.093) | 0.045*** (<0.001) | 1.046 (1.021-1.071) |
| Stroke | -10.39 | 0.032 (0.189) | 1.033 (0.984-1.084) | 0.051*** (<0.001) | 1.053 (1.036-1.070) |

***P<0.001;

*Note: participants who had serum sodium outside 135-146 mmol/l range and glucose higher than 126 mg/dL at visit 1 were excluded from the analysis*

**Supplemental Table 3. Estimation of decrease in burden of chronic age-dependent diseases in 70-85 years old people residing in US as a result of maintaining optimal hydration.** The calculations shown in the table estimate the number of people who would not develop the diseases if all people with average serum sodium 142mmol/l and higher would decrease their average serum sodium concentration to 140-141.5 mmol/l by modification of water and salt intake starting at middle age or earlier. *The estimations are made based on data from Atherosclerosis Risk in Communities (ARIC) study.*

| **Dementia** | **Heart Failure** | **Chronic Lung Disease** | **Chronic Kidney Disease** | **Diabetes** | **High Blood Pressure** | **Stroke** |
|---|---|---|---|---|---|---|
| *Prevalence of the diseases for 142-146mmol/l sodium group (ARIC study)* | | | | | | |
| 4.8% | 10.2% | 20.4% | 29.3% | 28.3% | 77.6% | 12.8% |
| *Prevalence for the diseases for 140-141.5mmol/l sodium group (ARIC study)* | | | | | | |
| 2.5% | 7.7% | 16.4% | 26.5% | 25.3% | 72.1% | 12.4% |
| *Potential decreases in prevalence of the diseases for people in 142-146mmol/l group if they reduce serum sodium to 140-14.51mmol/l at middle age by modifying water and salt intake (ARIC study)* | | | | | | |
| 48% | 24% | 20% | 10% | 11% | 7.1% | 3.1% |
| *Predicted number of people 70-85 years old, who would not develop the diseases (if extrapolated to whole US population: 28,000,000 70-85yeas old people)* | | | | | | |
| 342,000 | 363,000 | 597,000 | 422,000 | 442,000 | 822,000 | 59,000 |
| Total: 3,047,000 people | | | | | | |

Full unedited gels for Figure 2I and Supplemental Figure 3

vWF
(predicted MW 270kDA)

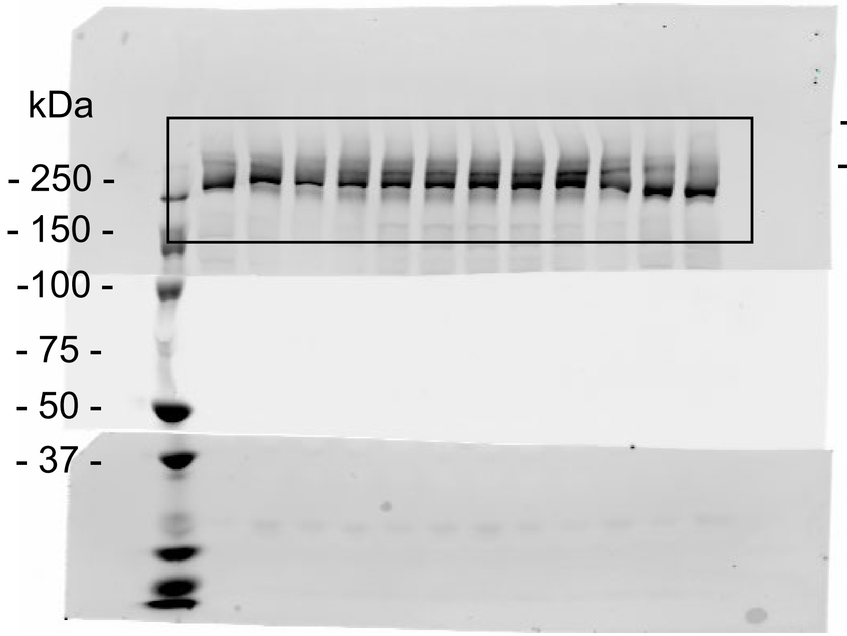


D-dimer
(predicted MW 340kDA)

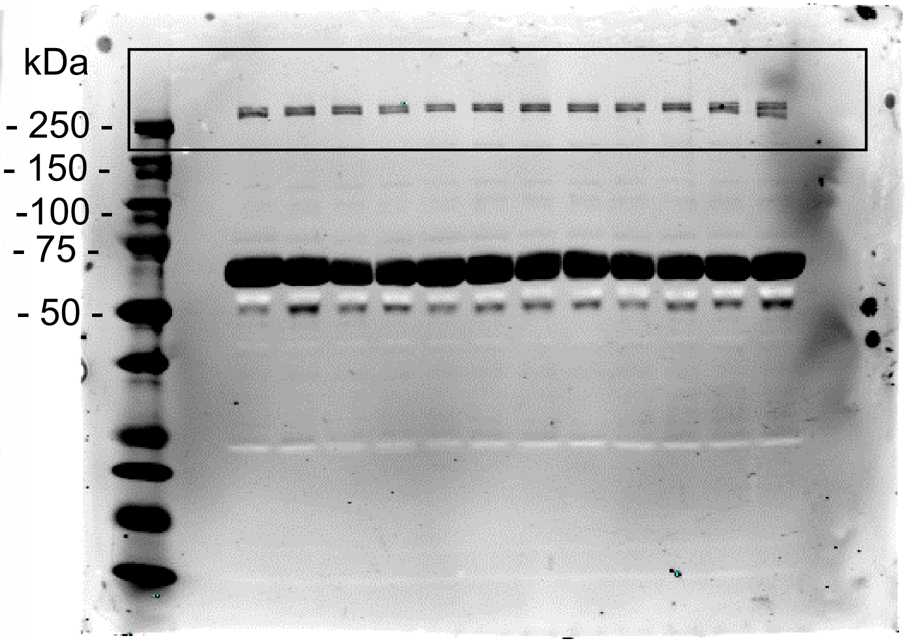


Note: this membrane was cut horizontally into 3 parts to probe for other proteins of different molecular weight. Only upper part was probed with vWF antibody
